# Memory function performance in individuals classified as overweight, obese, and normal weight

**DOI:** 10.3389/fnut.2022.932323

**Published:** 2022-11-21

**Authors:** Marina Berbegal, Mario Tomé, Miriam Sánchez-SanSegundo, Ana Zaragoza-Martí, José Antonio Hurtado-Sánchez

**Affiliations:** ^1^Department of Health Psychology, Faculty of Health Science, University of Alicante, Alicante, Spain; ^2^Department of Nursing, Faculty of Health Science University of Alicante, Alicante, Spain; ^3^Alicante Institute for Health and Biomedical Research (ISABIAL-FISABIO Foundation), Alicante, Spain

**Keywords:** overweight, obesity, normal weight, memory function, adiposity

## Abstract

Evidence accumulated to date about the relationship between cognitive impairments and adults who are overweight and obese suggests that excess weight has a great impact on memory function. Nevertheless, most of the literature has focused only on studying the influences on working memory and episodic memory. This study aimed to examine the potential associations of clinical and anthropometric measures [body mass index (BMI), WHR, body fat, visceral fat, muscle mass, and hypertension] with six memory domains, including contextual memory, short-term visual memory, short-term memory, non-verbal memory, short-term phonological memory, and working memory, in a sample of 124 individuals classified as overweight (*n* = 33), obese (*n* = 53), and normal weight (*n* = 38). The results obtained showed that, after controlling for employment situations, people classified as obese had poorer short-term phonological memory and working memory than those with normal weights. Bivariate correlations showed that measures of weight, BMI, waist–hip ratio index, body fat, and visceral fat were inversely associated with memory function. However, muscle mass was not a significant predictor of memory function. Higher systolic blood pressure was also associated with worse memory function. The study provides evidence of the importance of adiposity in health and memory function.

## Introduction

According to the World Health Organization (WHO) (1), overweight and obesity are conditions defined as an excess or abnormal accumulation of fat that can be harmful to health. Being overweight or obese depends on body mass index (BMI) classification, where a score of over 25 is considered overweight, and that over 30 is obese. The prevalence of overweight and obesity has tripled in the last three decades, turning into an important world health problem. In 2016, more than 1.900 billion adults were overweight and more than 650 million were obese (2). According to the European Health Interview Survey, the prevalence of overweight and obesity in Europe has increased dramatically over the past decades in many regions (3). In Spain, the proportions of men and women who were overweight were 44.9 and 30.6%, respectively, while the prevalence of obesity was 16.5% for males and 15.5% for females (4).

Epidemiological studies have shown a clear association between overweight and obesity and the occurrence of chronic diseases, such as diabetes mellitus (5), musculoskeletal disorders (6), high blood pressure, cardiovascular diseases (7), and cancer (8). Furthermore, it has been found that eating habits characterized by the intake of fat and refined foods not only have a negative impact on physical health but also contribute to greater cognitive decline and the appearance of neurodegenerative pathologies, including cognitive impairment and dementia (9–11).

Recent studies have shown a bidirectional relationship between obesity and cognitive function. Detrimental associations between anthropometric measures of obesity (e.g., BMI or waist circumference) and some cognitive domains were reported (12). Nevertheless, alterations in neuropsychological processes, such as poor performance in attention, memory, or executive functions, can also have an impact on behaviors prone in individuals with obesity (e.g., appetite dysregulation, decision-making, poor dietary choices, and a tendency toward uncontrolled eating) (13). The literature shows that memory is essential for food-related decision-making and has a great impact on appetite control and weight gain (13), especially working memory (14, 15) and episodic memory (16). This bidirectional relationship between obesity and cognitive function shows two pathways. The present study is focused on the first, which is the association between anthropometric measures of obesity and neurocognitive performance. Recent researchers have reported that cerebral inflammation produced by the accumulation of adipose tissue, activation of the immune system, and gray matter atrophy could be some of the mechanisms involved in this relationship (17–19).

In connection with these findings, executive functions and memory are two domains that are affected by a high level of BMI (15). The executive functions (EFs) constitute a set of cognitive capacities necessary to control and self-regulate an individual’s behavior (20). The EFs include cognitive processes, which are cognitive flexibility, monitoring, inhibition, planning, working memory, and processing speed (21). This set of cognitive abilities allows us to organize, integrate and manipulate information acquired, giving us the ability to make decisions, create, anticipate, and plan future goals (22). The literature has demonstrated that EFs can be affected by the accumulation of adipose tissue in different organs, tissues, and systems of the body (23). A previous meta-analysis of 72 studies demonstrated that higher BMI contributed to the appearance of deficits in the cognitive abilities of inhibition, decision-making, working memory, planning, and cognitive flexibility (23).

Furthermore, recent research on brain dysfunction in individuals who are overweight or obese reported that excess weight had a greater effect on memory function (13), which is a cognitive process through which information is encoded, stored, and retrieved (24). According to the literature, there exist several types of memory based on classification criteria. Regarding recall time, immediate memory, working memory, short-term memory, and long-term memory can be distinguished. However, referring to the voluntariness of memory, implicit memory, unconscious process memory, explicit memory, episodic, and semantic voluntary memory can be differentiated (25). The evidence indicates that each type of memory and each process that we use has neural activations associated with different brain areas. The medial temporal cortex, the hippocampus, the prefrontal cortex, and the cerebellum are some of the zones involved in memory function (26). For example, a study about neural activity in low- and high-BMI participants reported that individuals with higher BMI had poorer activity in memory structures than those with normal weights (27). A study by Prickett et al. (28) also found that obesity was predictive of poorer performances in verbal memory and working memory. Other neuropsychological studies have demonstrated lower performances in visual memory, prospective memory, and verbal memory (29). Nevertheless, to date, the majority of studies have focused on the study of episodic and working memory, neglecting other essential memory types for correct day-to-day performance.

Since the great influence of BMI on memory has been demonstrated, it is important to study how the effect of excess fat affects memory subcomponents. For this reason, to expand the spectrum of memory functions, the present study aims to analyze the potential associations between clinical and anthropometric measures and memory subcomponents (contextual memory, short-term visual memory, short-term memory, non-verbal memory, and short-term phonological memory) in individuals classified as overweight, obese, and normal weight. We hypothesize that higher clinical and anthropometric measures are associated with poorer memory subcomponents in individuals classified as obese and overweight compared to adults with normal weight.

## Materials and methods

### Participants and procedure

The sample included a total of 124 male and female Spanish participants between the ages of 22 and 63 (*M* = 46.02 years; SD = 9.31). The sample size was estimated with a power calculation using G*Power3 (30). Calculations of 80% power with an alpha of 0.05 suggested that 35 participants per group were needed to detect an effect with a medium effect size of 0.25. Participants were recruited by advertisements on the website of the Tech4Diet project: 4D modeling and visualization of the human body. The inclusion criteria were: (i) a BMI greater than 24.9 kg/m^2^ [overweight (25 ≥ BMI < 30) and obese (BMI ≥ 30)] or (ii) a BMI smaller than 24.9 (normal weight (18.5 ≤ BMI < 25), according to the BMI classification of the WHO; (iii) the ability to read and write fluently; and (iv) Spanish as a mother tongue. The exclusion criteria were (i) currently being or having been in dietetic–nutritional treatment supervised by a nutritionist in the last year; (ii) the presence of an endocrine–metabolic disorder (including thyroid, pituitary gland, and adrenal gland problems and metabolic syndrome); (iii) the presence of a previous history of neurological disease (e.g., stroke or Parkinson’s disease) or a history of head trauma; (iv) the presence of a history of severe psychopathology according to the diagnostic criteria of the DSM-IV-TR; and (v) currently receiving psychiatric treatment. Initial participants were recruited from September to November 2020. Normal-weight participants were recruited in June 2021. Of the 126 initial volunteers, two were excluded from the study for having histories of endocrine–metabolic disorder. The final sample included 124 male and female participants classified as overweight, obese, or normal weight. All the measurements were conducted on one testing day. Additionally, all the participants completed a neuropsychological battery of executive function tests. Data were collected at ALINUA, a nutrition and food cabinet endorsed as a health center dependent on the Faculty of Health Sciences of the University of Alicante. The duration of neuropsychology sessions lasted approximately 40 min.

### Ethical considerations

The study was approved by the Ethical Committee of the University of Alicante, as well as by the Ethics Committee of the Instituto de Investigación Sanitaria y Biomédica de Alicante [ISABIAL (Health and Biomedical Research Institute of Alicante)] (CEIm: 180380). The study is also part of two ongoing research projects funded by the Ministry of Science and Innovation: *“4D modeling and visualization of the human body for the improvement of adherence to dietary-nutritional treatment of obesity through low-cost technologies”* (TIN2017-89069-R) and *“Predictive models of the morphological evolution of the human body to improve adherence”* (PID2020-119144RB-100). After participants were informed about the voluntary nature of their participation and the fact that they could withdraw from the study whenever they wanted and without consequences, informed consent was obtained from all the subjects involved.

### Measures

#### Anthropometrics, body composition, and clinical parameters

A TANITA MC-780MA P digital weight scale (TANITA Corporation, Arlington Heights, IL, USA) and a 213 SECA portable stadiometer (SECA, Hamburg, Germany) were used to carry out the weight (0.1 kg precision), body fat (0.1 kg precision), visceral fat (cm), muscle mass (0.1 kg precision), and height (0.1 cm precision) measurements. Body mass index (BMI) was calculated as weight/height squared (kg/m^2^). According to the WHO classification, we established the cut-off point for overweight as 24.99 kg/m^2^, while obesity was defined as a BMI over 30 kg/m^2^ and normal weight as 18.5–24.99 kg/m^2^.

The waist and hip circumferences were measured using a flexible measuring tape (measurement precision, 0.1 cm). To ensure accurate results, all the measurements were performed twice, and the waist–hip ratio (WHR) value was calculated as the ratio of the waist to hip circumference.

Blood pressure (systolic and diastolic) was measured using an M7 Intelli IT blood pressure monitor (OMROM, M7, Corp., Kyoto, Japan). Capillary cholesterol (mg/dl), glucose (mg/dl), and triglyceride (mg/dl) concentrations were also examined with an Accutrend^®^Plus instrument using two drops of blood (15–40 μl) collected from different fingers with a lancing device (Accuchek^®^ Softclix^®^ Pro, Roche Diagnostics GmbH, Mannheim, Germany).

#### Memory (cognitive function)

Memory was examined using the CogniFit General Cognitive Assessment (CAB). It is a computer-assessed neuropsychological test battery commonly used in protocols of cognitive skills research. The CogniFit neuropsychological battery has been widely used for clinical and research purposes (31) since the tests that it offers have been validated against various standard neuropsychological tests (32). Furthermore, scientific studies using CogniFit activities in healthy children, adults, and older people with the aim of improving cognitive function are numerous and have high methodological quality, giving CogniFit the highest level of empirical evidence (31, 33, 34). CogniFit scores range from 0 to 800 points, where high scores refer to increased cognitive performance. For scores between 0 and 200 (red), cognitive abilities are considered cognitive weaknesses. Patients with scores of 200--400 (yellow) are considered patients with cognitive abilities within what is expected for people of their age and gender, but they are still improvable. Higher scores in the range of 400--600 (green) mean that cognitive abilities with these scores are in good condition. Cognitive abilities that show scores above 600 (green) are considered strengths or cognitive skills since they are in better condition than those of other people of the same sex and age. Scores on the six cognitive abilities^1^ are assigned using weights previously derived from factor analyses performed on normative data and are standardized into *Z*-scores. In the present study, we used the memory measures of the General Cognitive Assessment (CAB). Specifically, we examined the following: contextual memory (ability to memorize and discriminate the real source of a specific memory), short-term visual memory (ability to temporarily retain a small amount of visual information), short-term memory (ability to retain a small amount of information to be used in a short period of time), non-verbal memory (ability to store and retrieve non-verbal information by nature), short-term phonological memory (ability to remember phonological information for a short period of time) and working memory (ability to temporarily store and handle information in order to perform complex cognitive tasks). CogniFit offers a wide battery of exercises designed not only to evaluate cognitive function but also to rehabilitate problems in memory or other cognitive functions with practice and cognitive training.

The following are the names and descriptions of the tasks in the cognitive training program (CAB):

1. ***Numbers:*** A series of numbers is displayed, from 2 to 10 digits. The task consists of memorizing them to exactly reproduce them later. *Working memory, short-term memory, and short-term phonological memory.*
2. ***Three figures**:* Three figures are shown for a short period of time. Subsequently, four possible trios of figures are shown. The task is to select the one that corresponds exactly to the first sequence shown. *Working memory and non-verbal memory.*
3. ***Illuminated circles:*** Circles light up in a specific order. The task is to exactly memorize the order and execute it when it is the individual’s turn. *Short-term memory, working memory, non-verbal memory, and short-term visual memory.*
4. ***Objects seen or heard**:* This task requires sound. Objects are presented one after another. If it is the first time that an object appears as an image or its name is heard, the patient must press “not presented”. If the object was last presented as an image, the patient presses “presented as an image”, and if it was last heard, the patient presses “presented orally”. *Contextual memory, working memory, and non-verbal memory.*
5. ***Images and words**:* This task requires sound. For a short period of time, some objects appear one after another. Then some words appear, either written or heard. The task consists of determining whether they were previously displayed by pressing the appropriate button (presented or not presented). At the end of each level, the exercise asks the patient to estimate how many questions were answered correctly. *Contextual memory and non-verbal memory.*

### Data analysis

First, participants were classified into different groups considering their BMI. This classification was conducted following the BMI classification of the WHO. After group classification, differences in the prevalence of participants in each group depending on sociodemographic data (sex, marital status, educational level, and employment situation) were analyzed employing Chi-squared statistics, as well as ANOVA for age. Anthropometric, body composition, and clinical parameter comparisons between subjects classified as overweight, obese, and normal weight were evaluated using ANCOVAs and controlling for the effects of the employment situation. Furthermore, to identify the possible differences between groups in the different types of memory (contextual memory, short-term visual memory, short-term memory, non-verbal memory, short-term phonological memory, and working memory), ANCOVAs were conducted controlling for the effects of employment situation separately for each type of memory. Bonferroni correction was used in *post hoc* comparisons. A value of *p* < 0.05 was considered significant in all cases. Partial eta square was used as the effect size measure. Multiple clinical, anthropometric, and memory features were assessed, and Pearson’s correlation was used to explore possible correlations among them. All the statistical analyses were performed using SPSS, Version 24.0 (Armonk, NY, USA). The descriptive values were expressed as the mean and standard deviation (*M* and SD, respectively).

## Results

### Frequency and percentage of sociodemographic variables

The sociodemographic data are presented in Table 1. There were no significant differences between groups in sex, age, marital status, and educational level. The sample differed in terms of employment situation (*p* < 0.05), as shown in Table 1. Although small, the differences between groups in employment situation (*p* = 0.04) could be explained by the “unemployed” individuals, as in the obesity group there were 10, while in the others there were 0 and 2, showing a small difference between these groups.

**TABLE 1:** Frequency and percentage of sociodemographic characteristics for participants in each group.

		Obese (*n* = 53)	Overweight (*n* = 33)	Normal weight (n = 38)	*p*
Sex	Male	19 (39.6%)	12 (25.0%)	17 (35.4%)	0.65
	Female	34 (44.7%)	21 (27.6%)	21 (27.6%)	
Age		48.43 (8.37)	45.82 (9.52)	42.84 (9.63)	0.45
Marital status	Single	6 (30.0%)	6 (30.0%)	8 (40.0%)	0.75
	Married	41 (46.1%)	23 (25.8%)	25 (28.1%)	
	Divorced	6 (40.0%)	4 (26.7%)	5 (33.3%)	
	Other	0 (0.0%)	0 (0.0%)	0 (0.0%)	
Educational level	Non-studies/primary studies	6 (66.7%)	1 (11.1%)	2 (22.2%)	0.09
	Secondary studies	24 (54.5%)	9 (20.5%)	11 (25.0%)	
	University studies	23 (32.4%)	23 (32.4%)	25 (35.2%)	
Employment situation	Full-time/part-time job	43 (38.4%)	33 (29.5%)	36 (32.1%)	0.04*
	Unemployed	10 (83.3%)	0 (0.0%)	2 (16.7%)	
	Other	0 (0.0%)	0 (0.0%)	0 (0.0%)	

*p < 0.05.

### Bivariate correlations

Bivariate correlations among weight, height, BMI, WHR, body fat, visceral fat, muscle mass, systolic blood pressure, diastolic blood pressure, glucose, cholesterol, triglycerides, contextual memory, short-term visual memory, short-term memory, non-verbal memory, short-term phonological memory, and working memory are presented in Table 2. Significant correlations were found between weight and short-term memory (*p* = 0.03), non-verbal memory (*p* = 0.04), short-term phonological memory (*p* = 0.01), and working memory (*p* = 0.01). Height was associated with contextual memory (*p* < 0.01). In the case of BMI, significant correlations were found in short-term visual memory (*p* = 0.01), short-term memory (*p* < 0.01), non-verbal memory (*p* < 0.01), short-term phonological memory (*p* < 0.01) and working memory (*p* < 0.01). In WHR, correlations were associated with two memory types: short-term memory (*p* = 0.04) and working memory (*p* = 0.03). Body fat was found to have significant correlations with short-term memory (*p* < 0.01), non-verbal memory (*p* = 0.03), short-term phonological memory (*p* < 0.01), and working memory (*p* < 0.01). Visceral fat had significant correlations with short-term memory (*p* = 0.03), short-term phonological memory (*p* < 0.01), and working memory (*p* < 0.01). Finally, systolic blood pressure was associated with short-term visual memory (*p* = 0.03), short-term memory (*p* = 0.03), non-verbal memory (*p* = 0.01), and working memory (*p* = 0.01). The bivariate correlations were small or moderate, ranging from.18 to.34.

**TABLE 2:** Bivariate correlations between clinical and anthropometric measures and memory.

Variables	1	2	3	4	5	6	7	8	9	10	11	12	13	14	15	16	17	18
1. Weight (Kg)	1	0.411**	0.892**	0.586**	0.554**	0.770**	0.507**	0.504**	0.517**	0.064	0.012	0.134	0.012	–0.129	−0.186*	−0.185*	−0.217*	−0.231**
		0.000	0.000	0.000	0.000	0.000	0.000	0.000	0.000	0.479	0.891	0.137	0.893	0.154	0.039	0.040	0.015	0.010
2. Height (m)		1	–0.025	0.269**	−0.293**	0.181*	0.588**	0.222*	0.047	–0.048	–0.014	0.012	0.252**	0.103	0.134	0.112	0.136	0.150
			0.785	0.003	0.001	0.044	0.000	0.013	0.603	0.599	0.874	0.892	0.005	0.255	0.137	0.215	0.133	0.097
3. BMI			1	0.497**	0.751**	0.742**	0.272**	0.453**	0.545**	0.104	0.042	0.156	–0.129	−0.213*	−0.294**	−0.277**	−0.323**	−0.347**
				0.000	0.000	0.000	0.002	0.000	0.000	0.249	0.645	0.083	0.153	0.017	0.001	0.002	0.000	0.000
4. WHR				1	0.186*	0.626**	0.470**	0.545**	0.370**	0.163	–0.085	0.139	0.029	–0.152	−0.180*	–0.174	–0.139	−0.195*
					0.038	0.000	0.000	0.000	0.000	0.070	0.351	0.123	0.746	0.092	0.046	0.054	0.125	0.030
5. Body fat					1	0.494**	−0.203*	0.171	0.379**	0.060	0.095	0.097	–0.088	–0.148	−0.237**	−0.192*	−0.300**	−0.234**
						0.000	0.024	0.057	0.000	0.508	0.296	0.285	0.331	0.100	0.008	0.033	0.001	0.009
6. Visceral fat						1	0.296**	0.451**	0.438**	0.179*	0.059	0.129	–0.065	–0.110	−0.186*	–0.145	−0.242**	−0.292**
							0.001	0.000	0.000	0.047	0.517	0.152	0.475	0.225	0.039	0.108	0.007	0.001
7. Muscle mass (Kg)							1	0.384**	0.252**	0.067	–0.055	0.044	0.138	–0.010	–0.012	–0.002	–0.032	–0.044
								0.000	0.005	0.459	0.545	0.624	0.126	0.916	0.894	0.984	0.728	0.628
8. Systolic blood pressure								1	0.756**	0.258**	0.012	0.074	–0.042	−0.191*	−0.195*	−0.222*	–0.146	−0.229*
									0.000	0.004	0.892	0.416	0.645	0.034	0.030	0.013	0.106	0.010
9. Diastolic blood pressure									1	0.139	–0.019	0.101	0.111	–0.118	–0.151	–0.14	–0.157	–0.119
										0.122	0.837	0.264	0.219	0.192	0.095	0.121	0.081	0.188
10. Glucose										1	0.178*	0.264**	–0.122	–0.12	–0.143	–0.088	–0.101	–0.15
											0.048	0.003	0.178	0.185	0.112	0.330	0.265	0.095
11. Cholesterol											1	0.062	–0.032	0.120	0.107	0.168	0.027	0.024
												0.492	0.723	0.184	0.238	0.062	0.767	0.791
12. Triglycerides												1	0.006	0.035	0.057	0.06	0.057	0.016
													0.950	0.702	0.533	0.508	0.533	0.862
13. Contextual memory													1	0.389**	0.382**	0.556**	0.234**	0.797**
														0.000	0.000	0.000	0.009	0.000
14. Short-term visual memory														1	0.889**	0.904**	0.352**	0.569**
															0.000	0.000	0.000	0.000
15. Short-term memory															1	0.841**	0.719**	0.686**
																0.000	0.000	0.000
16. Non-verbal memory																1	0.390**	0.720**
																	0.000	0.000
17. Short-term phonological memory																	1	0.586**
																		0.000
18. Working memory																		1

*p < 0.05; **p < 0.01; ***p < 0.001.

### Differences in anthropometrics, body composition, and clinical parameters between individuals classified as overweight, obese and normal weight

Table 3 presents differences in anthropometrics, body composition, and clinical parameters among the participants. The obese group had higher weight [BMI: *F* (2) = 116.17; *p* < 0.01; η^2^ = 0.65], higher WHR [BMI: *F* (2) = 23.97; *p* < 0.01; η^2^ = 0.28], higher body fat [BMI: *F* (2) = 67.05; *p* < 0.01; η^2^ = 0.52], higher visceral fat [BMI: *F* (2) = 60.44; *p* < 0.01; η^2^ = 0.50], higher muscle mass (kg) [BMI: *F* (2) = 6.09; *p* < 0.01; η^2^ = 0.09], higher systolic blood pressure [BMI: *F* (2) = 21.24; *p* < 0.01; η^2^ = 0.26], and diastolic blood pressure [BMI: *F* (2) = 31.35; *p* < 0.01; η^2^ = 0.34] than those classified as overweight and normal weight. No significant intergroup differences were found in height or the clinical parameters of glucose, cholesterol, and triglycerides. The means and standard deviations for each group, as well as *post-hoc* analyses, are presented in Table 3.

**TABLE 3:** Means and standard deviations in clinical and anthropometric measures.

Variables	Obese (*n* = 53)	Overweight (*n* = 33)	Normal weight (*n* = 38)	*Post-hoc*	*p*	η*^2^*
			
Clinical measures	M (SD)	M (SD)	M (SD)			
Weight (kg)	99.28 (14.98)	76.94 (10.00)	62.74 (8.97)	OB > OV, N OB, OV > N OB > N	< 0.01***	0.65
Height (m)	166.47 (8.82)	166.06 (9.46)	166.32 (7.83)	OB > N, OV N > OV	0.51	0.01
WHR^1^	0.91 (0.10)	0.87 (0.08)	0.79 (0.08)	OB > OV, N OB, OV > N OB > N	< 0.01***	0.28
Body fat	38.21 (6.30)	30.99 (6.62)	21.12 (7.18)	OB > OV, N OB, OV > N OB > N	< 0.01***	0.52
Visceral fat	13.63 (4.99)	8.94 (4.10)	4.32 (2.49)	OB > OV, N OB, OV > N OB > N	< 0.01***	0.50
Muscle mass (Kg)	55.06 (9.34)	51.30 (8.96)	49.77 (10.03)	OB > OV, N OB, OV > N OB > N	< 0.01**	0.09
Systolic blood pressure	131.09 (13.81)	126.97 (16.30)	111.68 (15.48)	OB > OV, N OB, OV > N OB > N	< 0.01***	0.26
Diastolic blood pressure	86.60 (8.45)	82.15 (9.99)	71.94 (8.21)	OB > OV, N OB, OV > N OB > N	< 0.01***	0.34
Glucose	90.91 (14.60)	82.79 (20.86)	79.08 (21.08)	OB > OV, N OB, OV > N OB > N	0.69	0.00
Cholesterol	201.58 (31.57)	207.61 (38.79)	198.74 (43.06)	OV > OB, N OV > N	0.61	0.00
Triglycerides	262.28 (153.63)	235.00 (105.49)	228.84 (188.95)	OB > OV, N OB, OV > N OB > N	0.58	0.00

^1^WHR, waist–hip ratio. **p < 0.01; ***p < 0.001.

### Differences in memory between individuals classified as overweight, obese, and normal weight

Differences in the types of memory among participants classified as overweight, obese, and normal weight are presented in Table 4. There were no significant intergroup differences in contextual memory, short-term visual memory, short-term memory, and non-verbal memory. However, individuals with normal weights demonstrated better short-term phonological memory [BMI: *F* (3) = 6.00; *p* < 0.01; η^2^ = 0.09] and working memory [BMI: *F* (3) = 3.37; *p* = 0.02; η^2^ = 0.05] than those classified as obese and overweight. The means and standard deviations of each group for memory, as well as *post-hoc* analyses, are presented in Table 4.

**TABLE 4:** Means and standard deviations in memory subcomponents for each group.

Variables	Obese (*n* = 53)	Overweight (*n* = 33)	Normal weight (*n* = 38)	*Post-hoc*	*p*	η*^2^*
			
Memory	M (SD)	M (SD)	M (SD)			
Contextual memory	463.51 (189.38)	534.15 (164.43)	450.34 (154.96)	OV > OB, N OB > N	0.13	0.03
Short-term visual memory	394.87 (243.50)	448.88 (221.17)	442.16 (224.09)	OV > N, OB N > OB	0.48	0.01
Short-term memory	399.04 (223.51)	460.64 (177.59)	493.05 (189.80)	N > OV, OB N, OV > OB N > OB	0.08	0.04
Non-verbal memory	374.17 (180.93)	416.64 (162.08)	438.66 (168.67)	N > OV, OB N, OV > OB N > OB	0.26	0.02
Short-term phonological memory*	363.70 (181.82)	415.55 (160.62)	487.61 (157.81)	N > OV, OB N, OV > OB N > OB	< 0.01**	0.09
Working memory*	360.66 (161.25)	434.55 (127.84)	444.13 (132.43)	N > OV, OB N, OV > OB N > OB	0.02*	0.05

*M*, mean; SD, standard deviation; OV, overweight; OB, obesity; *N*, normal weight. *p < 0.05; **p < 0.01.

## Discussion

The present study aimed to identify the influence of clinical and anthropometric measures in six memory domains. In particular, we examined neuropsychological performances in contextual memory, short-term visual memory, short-term memory, non-verbal memory, short-term phonological memory, and working memory in adults classified as obese, overweight, and normal weight. Participants classified as overweight and obese performed with similar levels of cognitive functions in every type of memory domain. Participants with normal weight showed similar memory function results, with the exception of short-term phonological memory and working memory, for which those presenting obesity obtained worse scores.

Short-term phonological memory, which is one of the registers of sensorial memory, is defined as the ability to remember phonological information that we receive from an environment for a short period of time (35). It is registered in the primary auditory cortex, which is located in the temporal lobe; it is involved in auditory and language processing, and it is also responsible for memory functions and the management of emotions (35). The short-term phonological memory store extends to several brain areas, most of them located in the prefrontal cortex (PFC), since this is where executive control takes place and attentional control is monitored (36). Our results suggest that a higher BMI was related to lower memory performance in individuals who are obese. In addition, working memory, which is defined as the ability to temporarily store and handle the information to perform complex cognitive tasks, was found to be affected in those with higher BMIs. Studies with functional magnetic resonance imaging have shown how the dorsolateral prefrontal cortex plays an essential role in working memory. This area acts as a mediator between information from posterior sensory areas and the limbic system, thus integrating and providing feedback between sensory and emotional information with the purpose of organizing behavior to achieve a specific goal (37). The association between obesity and lower memory performance found in our study is in line with previous studies that have suggested that working memory is frequently affected by higher BMI (15, 23, 27). Some mechanisms may explain these results. In particular, recent research reported that the accumulation of adipose tissue as a result of being overweight and obesity produced chronic inflammation in organisms that were able to disrupt the structure of essential organs, such as the brain, producing a significant impact on cognitive functions (10). An increased BMI was associated with gray matter atrophy in the temporal, frontal, and occipital cortices, as well as the thalamus and midbrain (38). In particular, previous studies involving patients with dementia and laboratory studies in rodents have related obesity to structural and metabolic changes in the hippocampus (39–41), which is directly involved in memory processes. Furthermore, structural modifications in the prefrontal cortex caused by the activation of the immune system due to inflammatory processes were associated with working memory impairments (42). A recent study about eating behavior reported that lower working memory was associated with a loss of control in eating behavior and the choice of highly calorie-dense foods, particularly with higher snack food and fat intakes (43). Thus, a deficit in working memory may lead to impulsive, excessive, and less flexible eating behavior (44). However, there is limited literature about the implications of short-term phonological memory and BMI. A recent meta-analysis by Cheke et al. (27) found that the volume of the temporal lobe in patients with adiposity was lower due to cerebral atrophy (45, 46). Due to short-term phonological memory being located in the primary auditory cortex, a lower temporal lobe volume may lead individuals who are obese and overweight to worse short-term phonological memory performance.

The results of the present study suggest that weight, BMI, WHR index, body fat, and visceral fat were inversely associated with memory function. However, muscle mass was not a significant predictor of memory function. Evidence from independent studies showed a negative association between anthropometric measures (weight, BMI, and WHR) and cognitive performance (47). Recent cross-sectional evaluations have also demonstrated how increased WHR and visceral adiposity are associated with reduced cognitive scores (48–51). Studies in obese rodents and individuals have found considerable evidence for reduced memory performance (52, 53). In longitudinal studies of patients classified as normal weight, overweight, and obese, higher levels of BMI have been related to hippocampal atrophy and cortical thinning (54, 55). For example, Debette et al. (56) found that WHR was associated with changes in the total brain volume. Evidence also suggests that higher levels of body fat might produce adverse effects on health, including cognitive and neuroanatomical changes. A study by Nyberg et al. (53) with a sample of 581 healthy individuals found that higher body fat was negatively related to subcortical and hippocampal volume and memory. Furthermore, visceral fat is considered an important risk factor in the development of resistance to insulin and is present in various stages of obesity-induced hippocampal dysfunction, which is a brain area involved in memory processes (57). Research has demonstrated that memory processes are of critical importance, as they have a great impact on appetite regulation and weight gain (43). Memories of specific recent eating episodes play an important role in directing food choices and influencing when and how much a person eats. Interrupting memory processes may lead to overconsumption and obesity since it is the remembered experience rather than the actual experience that is more strongly associated with future choices (44). In the present study, WHR and visceral fat were as strong predictors as body fat for memory function. These results might indicate that visceral fat and WHR, apart from increasing the adipose tissue surrounding the intra-abdominal organs (58), might also have a significant impact on organs, such as the brain. There is consistent evidence that suggests body fat plays a more direct role in the brain, rather than visceral fat and WHR (15, 59). This suggests that it is important to study the adiposity continuum and to use complementary measures rather than only BMI. Interestingly, higher systolic blood pressure in participants was negatively associated with worse memory function. These results might indicate that hypertension, which has been associated with an increased risk of cognitive decline (60, 61), may play a direct role in memory. This phenomenon is thought to occur because hypertension disrupts the structure and function of cerebral blood vessels and leads to ischemic damage to white matter regions, which are critical for cognitive function (60). Evidence from previous studies found an association between hypertension and memory deficit (62).

There are several limitations in the current study that suggest areas for future research. First, the study was cross-sectional, precluding the establishment of causal inferences. Second, researchers must be careful when generalizing and interpreting the findings, as we used a small sample size from a single city in Spain, and the effect sizes were small. Third, the participants in our study were voluntarily recruited from the community; therefore, these individuals might be more highly motivated to lose weight and less resistant to change than the general community. Fourth, CogniFit evaluates cognitive domains that may not fit different memory classifications, neglecting memory subtypes that may also be relevant. The evaluation and analysis of memory functions entail great complexity since the domains of memory, while distinct, still share similar functional and structural pathways in the brain, and it is unclear why or how some domains are expected to differ while others are not. Further investigations are needed to clarify these questions, and it would be helpful to use additional neuropsychological tests to measure other interesting types of memory function. As suggested in previous studies, there is a bidirectional relationship between obesity and cognitive function since impaired cognition can hinder eating self-regulation and obesity can generate changes at the neurological level (14). Memory is a domain that can be affected by higher BMI, but it is also possible that deficits in memory may lead individuals to worse dietary choices. While looking for evidence, it was difficult to assess the relevance of previous neuroscientific findings to understanding short-term phonological memory function in obesity. Therefore, further investigations are needed to understand the neural mechanisms underlying short-term phonological memory deficits in obese individuals. Regarding the relationship between anthropometric measures (weight, BMI, body fat, WHR, and visceral fat) and memory, the literature shows diverse results, so deeper investigations are required. Furthermore, recent studies demonstrated that hypertension produced an increased risk of cognitive decline (61). However, there is still a lack of consistent findings on the impact of hypertension on memory function, so future studies should test this hypothesis. Finally, as far as we know, this is one of the first studies conducted in Spain assessing different types of memory function between individuals classified as overweight, obese, and normal weight. Despite these limitations, this study provides evidence of the importance of adiposity in health and memory function since the findings serve to strengthen this association, as well as claim and propose the importance of cognitive functions in clinical nutritional processes. The presence of a cognitive stimulation protocol that has the objective of preventing dysfunction in premature cases and recovering performance in more advanced cases is considered to be essential. For this reason, it would also be interesting to analyze whether, through an anti-inflammatory nutritional dietary protocol (e.g., the Mediterranean diet) (63), cognitive dysfunctions caused by obesity-induced inflammation can be reversed.

## Conclusion

The results provided evidence of the influence of anthropometric measures on memory function in individuals classified as obese, overweight, and normal weight. In particular, our findings suggested the importance of examining the independent roles of body fat, visceral fat, and WHR in the memory function of participants considered overweight and obese, since it has been demonstrated that the accumulation of fat in different regions of the body might suggest different memory impairments.

## Data availability statement

The original contributions presented in this study are included in the article/supplementary material, further inquiries can be directed to the corresponding authors.

## Ethics statement

This study was approved by the Ethical Committee of the University of Alicante, and also by the Ethics Committee of the Instituto de Investigación Sanitaria y Biomédica de Alicante [ISABIAL (Health and Biomedical Research Institute of Alicante)] (CEIm: 180380). The patients/participants provided their written informed consent to participate in this study.

## Author contributions

MB: conceptualization, formal analysis, data curation, and writing—original draft preparation. MB, MS-S, AZ-M, and MT: methodology and writing—review and editing. MB, MT, MS-S, AZ-M, and JH-S: investigation. MB and MT: resources. JH-S: supervision. MS-S and AZ-M: funding acquisition. All authors have read and agreed to the published version of the manuscript.

## Footnotes

1 http://www.cognifit.com (accessed on 30 December 2021).
